# Effect of Calcium Silicate-Based Intracanal Medicament and Calcium Hydroxide on Growth Factor TGF-β1 Release from Root Canal Dentine

**DOI:** 10.3390/jfb15060139

**Published:** 2024-05-22

**Authors:** Goda Bilvinaite, Saulius Drukteinis, Egidijus Simoliunas, Matthias Widbiller, Simas Sakirzanovas

**Affiliations:** 1 Department of Applied Chemistry, Institute of Chemistry, Vilnius University, Naugarduko 24, LT-03225 Vilnius, Lithuania; goda.bilvinaite@mf.stud.vu.lt (G.B.); simas.sakirzanovas@chf.vu.lt (S.S.); 2 Institute of Dentistry, Faculty of Medicine, Vilnius University, Zalgirio 115, LT-08217 Vilnius, Lithuania; 3 Department of Biological Models, Life Science Center, Institute of Biochemistry, Vilnius University, Sauletekio al. 7, LT-10257 Vilnius, Lithuania; egidijus.simoliunas@gmc.vu.lt; 4 Department of Conservative Dentistry and Periodontology, University Hospital Regensburg, Franz-Josef-Strauß-Allee 11, D-93093 Regensburg, Germany; matthias.widbiller@ukr.de

**Keywords:** dentin, calcium hydroxide, calcium silicate, regenerative endodontics, TGF-β1

## Abstract

Effective root canal disinfection and the subsequent release of natural growth factors from dentin are crucial to the success of regenerative endodontic procedures. This study evaluated the effect of newly introduced calcium silicate-based temporary intracanal medicament Bio-C Temp and calcium hydroxide-based material UltraCal XS on the release of transforming growth factor β1 (TGF-β1) from root canal dentin. Twenty-two intact and fully developed human premolars from patients aged 15–18 were shaped and irrigated according to the current clinical recommendations. The teeth were then gently split in half, and the root canal dentin of paired samples was covered with Bio-C Temp or UltraCal XS. After 3 weeks of incubation, the specimens were conditioned with 17% EDTA and the collected solution was subjected to the quantification of the released TGF-β1 by performing an ELISA. One-way analysis of variance (ANOVA), followed by Tukey’s test, was selected to determine the statistically significant differences between the groups at the 0.95 confidence level. The highest mean value of released TGF-β1 (1993.1 pg/mL) was detected in the control group, where the root canal dentin was conditioned with 17% EDTA alone. Regarding the experimental groups, Bio-C Temp released a statistically significantly higher amount of TGF-β1 (282.14 pg/mL) compared to UltraCal XS (114.28 pg/mL; *p* = 0.0158). Bio-C Temp affected the release of growth factors from root canal dentin less than UltraCal XS and may therefore serve as an intracanal medicament for regenerative endodontic procedures.

## 1. Introduction

Traditionally, a root canal treatment is indicated when the pulp tissue becomes severely inflamed or necrotic due to caries, dental trauma or developmental aberrations. During the endodontic treatment procedure, the root canals are mechanically shaped, disinfected with appropriate irrigating solutions and filled with synthetic obturation materials, resulting in the total loss of all pulp functions [1]. This situation can be highly detrimental to immature permanent teeth, as pulp tissue removal at an early stage of root development prevents subsequent root maturation and apex closure [1,2]. Conventional endodontic treatment leaves immature permanent teeth with short roots, thin dentinal walls and an increased risk of root fracture in the long term [2,3]. Although implant therapy is now considered a safe and reliable method of replacing missing teeth, it can only be considered after the completion of growth in early adulthood and has a lower survival rate than natural teeth [3]. Therefore, attempts to preserve the natural teeth and improve their long-term prognosis have led to the development of regenerative endodontic treatment approaches. The new treatment strategy focuses on three main goals: (I) the resolution of clinical symptoms and periapical bone destruction and the restoration of normal tooth function; (II) the continuation and completion of root development; and (III) the regeneration of pulp or pulp-like tissue [1].

It should be highlighted that many terms have been recently used in the scientific literature to describe regenerative endodontic treatment strategies, including “pulp revascularization” or “revitalization” [4,5]. However, these terms mainly refer to the regeneration of pulp blood vessels and reestablishment of the blood flow, whereas the main idea of regenerative endodontics is to regenerate the functionality of the entire pulp–dentin complex [1,5]. Therefore, the term “regenerative endodontic procedures” (REP) is more precise and better reveals the concept of pulp tissue engineering. This term was included in the glossary of endodontic terms by the American Association of Endodontists (AAE) in 2020 and has been most commonly used ever since in scientific articles investigating the biologically based treatment approach [1].

Biological elements required for the success of regenerative endodontic procedures are based on a triad of endogenously or exogenously derived stem cells, scaffolds and growth factors [2]. The most clinically used approach is tooth revitalization, where the scaffold is a blood clot generated by inducing apical bleeding into the root canal space. This host-derived cross-linked fibrin network serves as a scaffold for cell homing. During bleeding, various types of cells, including stem cells, mainly originating from the apical papilla and periodontal ligament, migrate into the root canal space [1,4]. Pulp tissue regeneration highly depends on the survival, attachment, proliferation and differentiation of the recruited stem cells, which are controlled by signaling molecules and growth factors [5,6]. Thus, proper root canal disinfection and the release of endogenous growth factors from the root canal dentin play a decisive role in the success of regenerative endodontic procedures [4].

The dentin matrix has been reported to contain a variety of entrapped growth factors, such as transforming growth factor-β (TGF-β), fibroblast growth factors (FGF), bone morphogenetic proteins (BMP), vascular endothelial growth factors (VEGF), insulin-like growth factors (IGF), etc. [4]. These bioactive signaling molecules bind to the transmembrane or intracellular receptors in a target cell and induce specific cellular responses, e.g., migration, proliferation, differentiation or apoptosis [1]. Special attention is usually paid to TGF-β1, which increases the collagen secretion by dental pulp fibroblasts and is highly necessary for odontoblast differentiation and mineralization [2]. The release of this growth factor, which is embedded in the extracellular matrix of dentin, is mainly achieved by applying conventional chelating agents, such as ethylenediaminetetraacetic acid (EDTA) or citric acid [4,7]. However, various intracanal medicaments used between visits to improve the disinfection of the entire root canal system may also release bioactive molecules from dentin [4]. Calcium hydroxide paste, which has been the main intracanal dressing material applied in endodontics for decades [8], was shown to allow a higher amount of TGF-β1 release compared to EDTA alone [4]. Therefore, using calcium hydroxide paste in regenerative endodontic procedures appears to be preferable regarding root canal decontamination and subsequent growth factor release.

Various medications have been proposed and used for the disinfection of root canals in regenerative endodontic procedures, such as calcium hydroxide paste, triple antibiotic paste (TAP) in various combinations and double antibiotic paste (DAP) [1,5]. Nevertheless, calcium hydroxide has limitations, of which the main one is cytotoxicity [9]. When calcium hydroxide paste is accidentally extruded beyond the apex, the release of reactive hydroxide causes tissue necrosis [10,11]. This scenario can be extremely detrimental in regenerative endodontic procedures, owing to the compromised viability of stem cells, which typically reside near the root apex and guide the root formation process jointly with Hertwig’s epithelial root sheath [11,12]. Unfortunately, younger patients with immature teeth, the primary receivers of regenerative endodontic procedures [13], are directly associated with a higher risk of inadvertent extrusion due to the limited patient cooperation and open root apices [14]. Moreover, the complete removal of calcium hydroxide paste from various root canal irregularities has also been reported to be difficult, resulting in the blockage of the dentinal tubules due to the remnants on the root canal surface [15]. Another possible intracanal medicament for regenerative endodontic procedures is triple antibiotic paste (TAP) mixed with 1:1:1 ciprofloxacin, metronidazole and minocycline [11]. Although the paste is highly effective in disinfecting necrotic root canals, it has several disadvantages over calcium hydroxide paste, including discoloration, higher cytotoxicity to stem cells at higher concentrations, the lower promotion of growth factor release and even more difficult removal from root canals [4,16]. To avoid tooth discoloration due to the minocycline component, DAP without minocycline and the replacement of minocycline with other antibiotics, such as clindamycin and cefaclor, have been suggested as potential alternatives [16]. 

Recently, the first temporary, ready-to-use calcium silicate-based intracanal medicament, Bio-C Temp (Angelus, Londrina, Brazil), has been introduced to the market, opening up new perspectives for the potential improvement of root canal disinfection and conditioning between appointments. The Bio-C Temp paste is composed of glycol salicylate, titanium oxide, calcium tungsten, calcium aluminate, calcium oxide and calcium silicate [17]. The base resin is a vehicle for the paste that does not allow the complete hydration of the material, so the paste does not harden in the root canals, as with the majority of hydraulic calcium silicate-based endodontic materials, when delivered or release Ca^2+^ and OH^−^ ions [17]. Due to its properties and stability, Bio-C Temp can serve as an intracanal medication, eliminating the necessity for repeated applications, which gives it a potential advantage over calcium hydroxide-based medications that need frequent replacement [18]. 

The concept of using hydraulic calcium silicate-based materials is not new, as these permanent filling materials have already been successfully applied in various intracoronal, intraradicular or extraradicular endodontic procedures, such as vital pulp therapy, root canal obturation, apical plugs in teeth with open apices, the management of endodontic complications or retrograde fillings in endodontic surgery [16]. According to the manufacturer’s recommendations, the Bio-C Temp paste is suitable for application in teeth with incomplete root formation, perforations and external and internal root resorption before using root repair and filling materials. Additionally, the material has been recommended for pulpotomy procedures to stimulate the formation of a calcified barrier and endodontic regeneration treatment [17,18]. Although evidence-based data on Bio-C Temp’s physical, chemical and biological properties are still limited, initial studies have shown that Bio-C Temp is more biocompatible and easier to remove from the root canals than conventional calcium hydroxide paste [18]. Based on these observations, it has been speculated that a calcium silicate-based intracanal medicament may be a particularly attractive alternative to calcium hydroxide paste in regenerative endodontic procedures.

However, the applicability of calcium silicate-based intracanal medicaments in regenerative endodontic procedures has not been investigated. Thus, it is important to determine whether Bio-C Temp induces the release of growth factors from root canal dentin. Since the growth factor TGF-β1 has been reported to be essential for dentinogenesis, dentin repair and related tissue regeneration processes [2], the present study aimed to assess and compare the effects of calcium silicate-based temporary intracanal medicament Bio-C Temp and calcium hydroxide-based material UltraCal XS (Ultradent Products Inc., South Jordan, UT, USA) on TGF-β1 release from root canal dentin.

## 2. Materials and Methods

### 2.1. Teeth Selection

**Figure 1:**
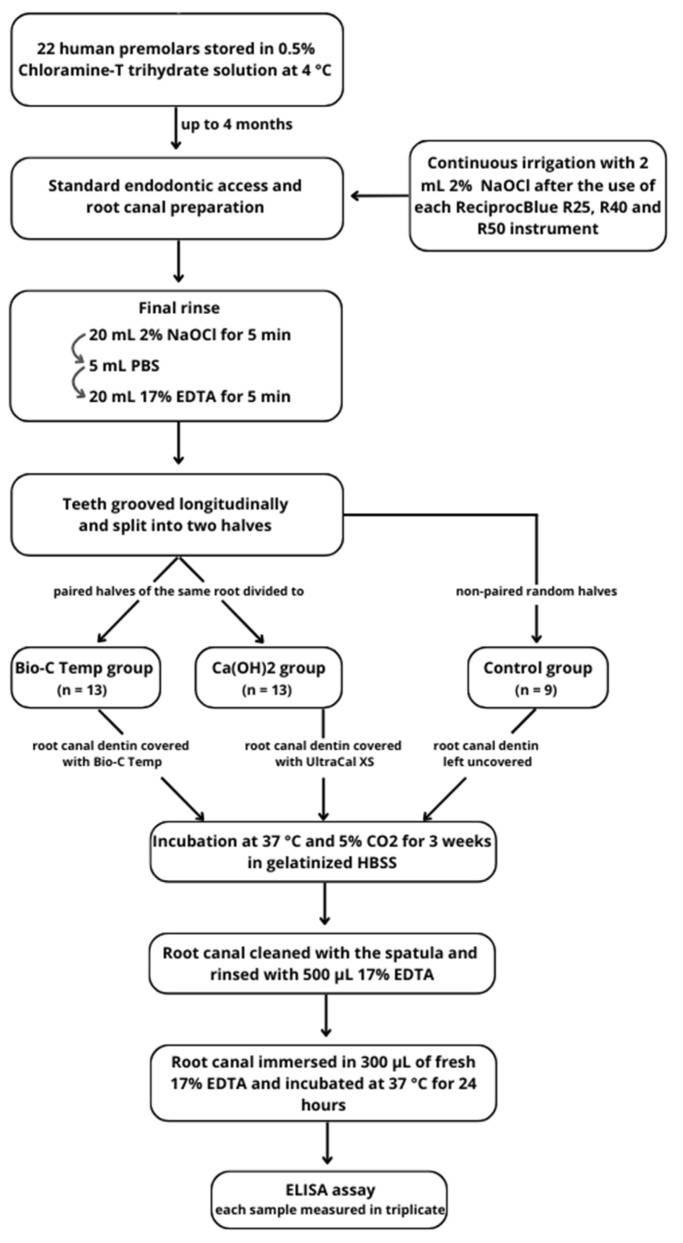
Flowchart of the study design.

The main aspects of the methodology are summarized in Figure 1. Twenty-two intact and fully developed human premolars from patients aged 15–18 years were collected under written patient consent and the approval of Lithuania’s national biomedical research ethics committee (protocol no. 158200-16-860-369).

Vital teeth were freshly extracted for reasons unrelated to the study, cleaned with periodontal curettes to remove soft tissue, rinsed with sterile phosphate-buffered saline (PBS) and stored in 0.5% Chloramine-T trihydrate solution at 4 °C for up to 4 months. Prior to tooth preparation, the storage solution was changed to distilled water and left for 24 h at room temperature. Only single-rooted premolars with a single root canal on preoperative radiographs and no anatomical malformations were selected.

### 2.2. Preparation and Conditioning of Root Canal Dentin

Standard endodontic access cavities were prepared using high-speed Endo Access burs (Dentsply Sirona, Ballaiques, Switzerland) under water cooling. The root canal working length (WL) was established by inserting a size 10 K-file (Dentsply Sirona, Ballaiques, Switzerland) into the canal until the tip was visible at the apical foramina. Subsequently, the instrument was retracted by 1 mm, and the WL was measured using an endodontic ruler. The mean WL of the samples was 19.86 ± 1.25 mm. The teeth were then fixed in prefabricated A-silicone blocks (3M ESPE, Seefeld, Germany) up to the cement–enamel junction to mimic the surrounding tissue and prevent the irrigating solution from overflowing beyond the apex.

Root canal shaping was performed with ReciprocBlue instruments (VDW, Munich, Germany) at the determined working length in the following sequence: R25 (25/0.08), R40 (40/0.06), R50 (50/0.05). After the use of each instrument, the root canals were repeatedly irrigated with 2 mL 2% sodium hypochlorite (NaOCl; Cerkamed, Stalowa Wola, Poland) using 29-G NaviTip needles (Ultradent Products Inc., South Jordan, UT, USA) attached to disposable syringes. The final rinse of the root canals at the end of mechanical preparation was performed as follows: 20 mL 2% NaOCl for 5 min, 5 mL PBS, 20 mL 17% EDTA (Cerkamed, Stalowa Wola, Poland) for 5 min. The shaped and cleaned root canals were dried with sterile paper points.

**Figure 2:**
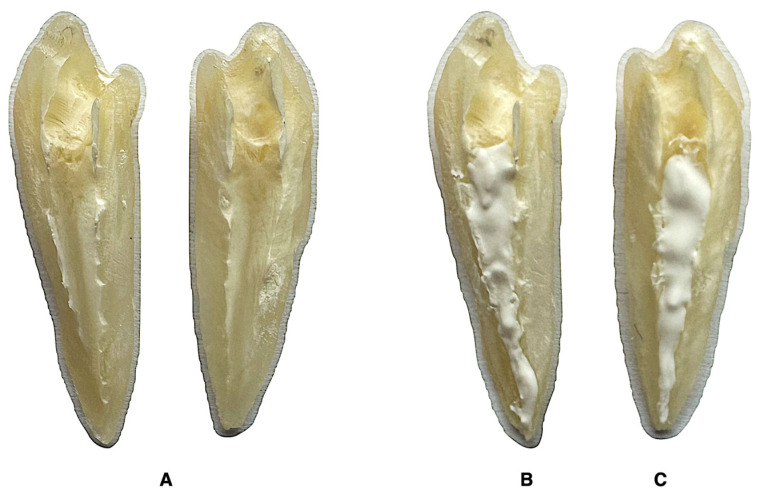
Paired halves of the mandibular premolar used for the experiment (**A**). Root canal dentin of the paired halves covered with Bio-C Temp (**B**) and UltraCal XS (**C**) intracanal medicaments.

The minimum sample size was calculated using the G*Power v.3.1 software (Heinrich Heine, Dusseldorf, Germany) with an α error probability of 0.05 and 1-β error probability of 0.80. A required size of 12 specimens per group was determined. Subsequently, all specimens were carefully grooved longitudinally on the buccal and lingual surfaces without penetrating the root canal. The teeth were gently split into two halves using a small chisel. The paired halves of the same root were randomly divided into two experimental groups, according to the intracanal medicament used (n = 13 per group) (Figure 2):

Bio-C Temp—the root canal dentin was covered with 0.05 ± 0.01 g calcium silicate-based paste Bio-C Temp.

Ca(OH)_2_—the root canal dentin of the paired half was covered with 0.05 ± 0.01 g calcium hydroxide paste UltraCal XS.

The control group consisted of 9 randomly selected halves that were not paired with the experimental groups. After splitting the teeth, the control root canal dentin was left untreated.

The specimens from all three groups were incubated separately in tissue culture dishes at 37 °C and 5% CO_2_ for 3 weeks. The external surface of each root half was immersed in gelatinized Hank’s balanced salt solution (HBSS) to avoid the dehydration of the specimen, while the internal surface was left uncovered, attempting to avoid material washout.

After the specified time, the bulk of the intracanal medicament was carefully removed with a spatula and the root canal dentin was rinsed with 500 μL 17% EDTA. The internal remnant-free root surface was then fully immersed in 300 μL of fresh 17% EDTA and incubated at 37 °C for 24 h. The EDTA’s contact with the external root surface was avoided. The collected solution was immediately frozen at −80 °C until further use.

### 2.3. Quantification of TGF-β1 Release

The thawed samples were subjected to the quantification of the released TGF-β1 by performing an ELISA assay. Each sample was measured in triplicate. Quantification was performed according to the protocol provided by the manufacturer of the selected ELISA test system (TGF-β1 Human ELISA Kit; Thermo Fisher Scientific, Waltham, MA, USA). The resulting absorbance values were within the range of the standard supplied with the ELISA kit.

### 2.4. Statistical Analysis

Statistical analysis was performed using the RStudio software v.4.3.1 (RStudio Inc., Boston, MA, USA). The assumption of normality was assessed by using the Shapiro–Wilk test and Q–Q plots. Since the Bio-C Temp group showed a non-normal distribution among the TGF-β1 measurements, the logarithmic transformation was applied. The assumption of normality was then accepted, and Levene’s test confirmed the homogeneity of variances. One-way analysis of variance (ANOVA), followed by Tukey’s test, was selected to determine the statistically significant differences between the groups at the 0.95 confidence level. The data are presented as the mean ± 95% confidence interval with a logarithmic scale in the y-axis.

## 3. Results

**Figure 3:**
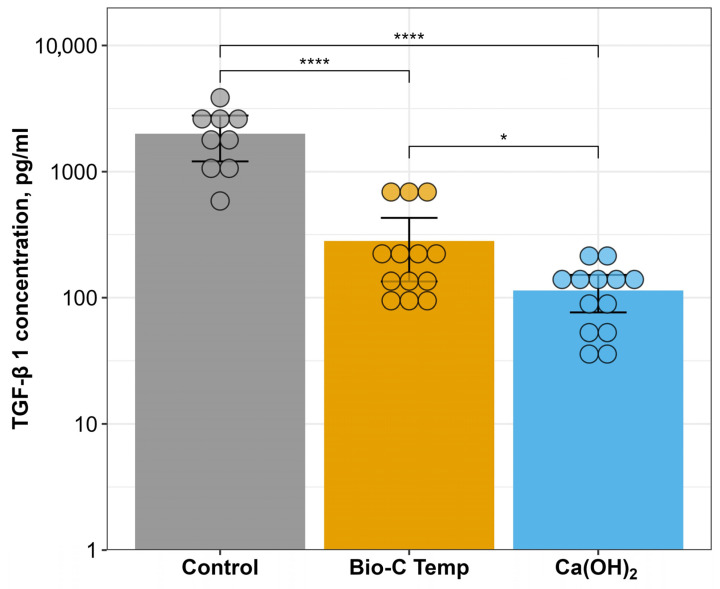
Concentrations of released TGF-β1 from root canal dentin treated with different intracanal medicaments for 3 weeks and subsequently conditioned with 17% EDTA. Data are presented as mean ± 95% CI with a logarithmic scale in the y-axis. Column height—mean of the group, error bars—95% CI, dots—individual tooth TGF-β1 value. * and **** indicate significant differences between the marked test groups at *p* < 0.05 and *p* < 0.001, respectively.

The ELISA measurements of the TGF-β1 release from the root canal dentin after the use of the calcium silicate-based temporary intracanal medicament Bio-C Temp and calcium hydroxide paste are summarized in Figure 3. The lowest concentration of released TGF-β1 was determined for the Ca(OH)_2_ group (mean = 114.28 pg/mL, 95% CI ± 37.64). The calcium silicate-based intracanal medicament Bio-C Temp resulted in higher growth factor release (mean = 282.14 pg/mL, 95% CI ± 147.52), and the difference between the Bio-C Temp and Ca(OH)_2_ groups was statistically significant (*p* = 0.0158). Nevertheless, the highest TGF-β1 concentration was determined for the control group (mean = 1993.1 pg/mL, 95% CI ± 790.10), indicating that both tested intracanal medicaments significantly reduced the amount of released growth factors from the root canal dentin.

## 4. Discussion

The present study aimed to investigate the effects of two different temporary intracanal disinfecting medicaments—calcium silicate-based material Bio-C Temp and calcium hydroxide paste—on transforming growth factor β1 release from root dentin compared to that in non-medicated samples. The results clearly demonstrated that both tested intracanal materials interfered with growth factor release. The calcium silicate-based temporary material Bio-C Temp promoted the significantly higher release of TGF-β1 than calcium hydroxide paste. Therefore, the null hypothesis tested was rejected.

Dentin is a complex structure composed of 70–72% inorganic and 20% organic components. The organic material is mainly type 1 collagen (90%), and the remaining part (10%) is dentin-specific proteins [19]. Among these specific proteins, dentin contains a wide variety of entrapped growth factors, which can modulate the recruitment, proliferation and differentiation of cells at very low concentrations and therefore play an indispensable role in regenerative endodontic procedures [20,21]. Although published studies continue to present various strategies for growth factor delivery systems relevant to dentin–pulp engineering, none of these efforts have reached clinical application yet [22,23]. Therefore, the dentin matrix, containing a plethora of growth factors, remains the main clinically available source of the required signaling molecules for regenerative endodontic approaches such as revitalization. According to the position statement by the European Society of Endodontology, 1.5–3% NaOCl irrigation followed by a rinse with a chelating solution should be used as a standard irrigation protocol for regenerative endodontic procedures [24]. Since numerous studies have demonstrated the ability of various organic acids to demineralize dentin and promote the release of embedded bioactive components [4,12,25], the current clinical recommendations for regeneration procedures suggest the use of 17% EDTA irrigating solution as the final rinse, which facilitates the exposure of entrapped growth factors and subsequentially induces the differentiation of blood clot-derived stem cells [20]. However, the application of intracanal medicaments between appointments may also affect the release of growth factors; thus, the amount of bioactive molecules liberated from the root canal dentin is highly dependent on the synergistic effect of the irrigating solution and the intracanal dressing materials used in the regenerative endodontic procedure [4,25]. These observations are reinforced by the results of our study, as both intracanal medicaments tested had an effect on the amount of TGF-β1 released. Despite the same irrigation protocol being used in the groups, the calcium silicate-based temporary material Bio-C Temp promoted the significantly higher release of growth factors than the calcium hydroxide paste, which is the main intracanal dressing material currently used in endodontic clinical practice.

However, the highest amount of released TGF-β1 was detected in the control group, where the root canal dentin was conditioned with 17% EDTA alone and no medicament was placed. This finding is consistent with the well-documented ability of EDTA to extract signaling molecules via the demineralization of the dentin extracellular matrix [2,20]. Interestingly, the combined effect of EDTA and the intracanal medicaments reduced the liberated amounts of TGF-β1, contradicting some previous studies in which calcium hydroxide paste did not affect TGF-β1 release as compared to EDTA alone [4]. One of the possible explanations could be related to the different incubation times of the specimens. As the short-term application of intracanal medicaments is not recommended or supported by any current clinical regenerative endodontic procedures or protocols [16], our study followed the statements and incubated samples for 3 weeks, whereas 48 h was chosen in the experiment of Galler et al. [4]. However, it should be mentioned that a longer incubation time may result in the deeper penetration of the intracanal medicaments into the dentinal tubules and make their further removal more difficult, thereby limiting the release of growth factors due to the physical barrier created by the medicament residues [16].

Another point to highlight is the intracanal medicament-initiated denaturation of the superficial dentin proteins over the longer incubation time. It has been demonstrated that both of the tested medicaments typically raise the pH up to 10.79 (Bio-C Temp) and 11.01 (Ultracal XS), which may induce the denaturation of dentin’s organic components and thus create superficial obliteration zones, limiting the release of growth factors that are entrapped in the deeper dentin layers. Even though various chelating agents, such as EDTA, are well known for their ability to debride the surfaces of the root canals and open the blocked dentin tubules, a prolonged period of irrigation and a higher amount of chelating agent is typically suggested in order to ensure both mechanisms—the debridement of the root canal and then the release of the entrapped growth factors. However, a relatively small volume of 17% EDTA was used in the present study, attempting to limit the dilution of the samples and to obtain traceable concentrations of TGF-β1 for the ELISA measurements. Therefore, the selected volume of the EDTA irrigating solution may have been insufficient to fully debride the conditioned root canal surfaces, and thus a higher amount of released growth factors was obtained in the non-medicated control group. Considering these limitations of the present study, the obtained differences between the control and experimental groups should be evaluated with caution. However, it should be also highlighted that the use of intracanal medicaments is unavoidable, as root canal disinfection is crucial for the survival of the delivered stem cells and, thus, for the overall success of regenerative endodontic procedures [16,26]. Therefore, based on the solid scientific evidence for the use of disinfecting intracanal agents between appointments and the previously confirmed acceptable biological properties of Bio-C Temp [18,27], our results suggest that the newly introduced calcium silicate-based temporary intracanal material has more potential for use in regenerative endodontic procedures than calcium hydroxide paste. Since it has been demonstrated that the ultrasonic activation of EDTA significantly enhances the growth factor release from the dentin due to the improved root canal debridement and ingression of the irrigating solution into the tubules [28], there is a possibility that even higher concentrations of TGF-β1 could be obtained in clinical use after the addition of various irrigant activation systems. However, to the best of our knowledge, the ability of Bio-C Temp to influence or modulate the release of growth factor TGF-β1 has not been investigated previously and, thus, the obtained results cannot be compared with other studies due to the lack of data.

Despite the advantageous aspects of our study, such as the paired samples and the organotypic experimental model of intact human premolars with a narrow age range (the quantity of growth factors in dentin may depend on the donor age due to the calcification processes taking place during the life span [28]), further experimental in vitro investigations and well-designed clinical trials are warranted to explore the broader implications of Bio-C Temp’s use in regenerative endodontic procedures. Understanding the effects of this new disinfecting material on stem cells and growth factors is critical to advancing in the field of revitalization procedures and translating these findings into practical clinical protocols.

## 5. Conclusions

In conclusion, the findings of this study indicate that dentin conditioning solely with 17% EDTA proved to be the most effective method in enhancing the release of growth factor TGF-β1, surpassing the capabilities of 17% EDTA combined with the temporary intracanal medicaments examined. Nevertheless, the calcium silicate-based material Bio-C Temp exhibited a higher concentration of released growth factor TGF-β1 compared to calcium hydroxide paste, demonstrating its potential to be used as an alternative for root canal disinfection in regenerative endodontic procedures. This suggests the need for the further investigation of its applicability in regenerative endodontic procedures.

## Data Availability

Data are contained within the article.
